# A case of bullous pemphigoid provoked by doxycycline-induced phototoxicity

**DOI:** 10.1016/j.jdcr.2023.05.005

**Published:** 2023-05-15

**Authors:** Isabella I. Sanchez, Katerina Yale, Bonnie A. Lee

**Affiliations:** a School of Medicine, University of California, Irvine, Irvine, California; b Department of Dermatology, University of California, Irvine, Irvine, California

**Keywords:** bullous pemphigoid, doxycycline, phototoxicity

## Introduction

Bullous pemphigoid (BP) is an autoimmune subepidermal blistering disease caused by autoantibodies directed against BP antigen 180 or BP antigen 230.^1^ BP is histopathologically characterized by a superficial dermal eosinophil-rich infiltrate with dermo-epidermal junction (DEJ) detachment. On direct immunofluorescence linear deposition of immunoglobulin G (IgG) and/or complement component 3 (C3) is present at the DEJ. Predisposing factors are broad and include human leukocyte antigen genes, aging, and trigger factors such as drugs, ultraviolet (UV) radiation, trauma, surgical procedures, and infections.^2^ Recognition of possible triggering factors is key and prompt removal can significantly improve prognosis. Here, we discuss an unusual case of new-onset BP in the setting of doxycycline-induced phototoxicity.

## Case report

**Fig 1:**
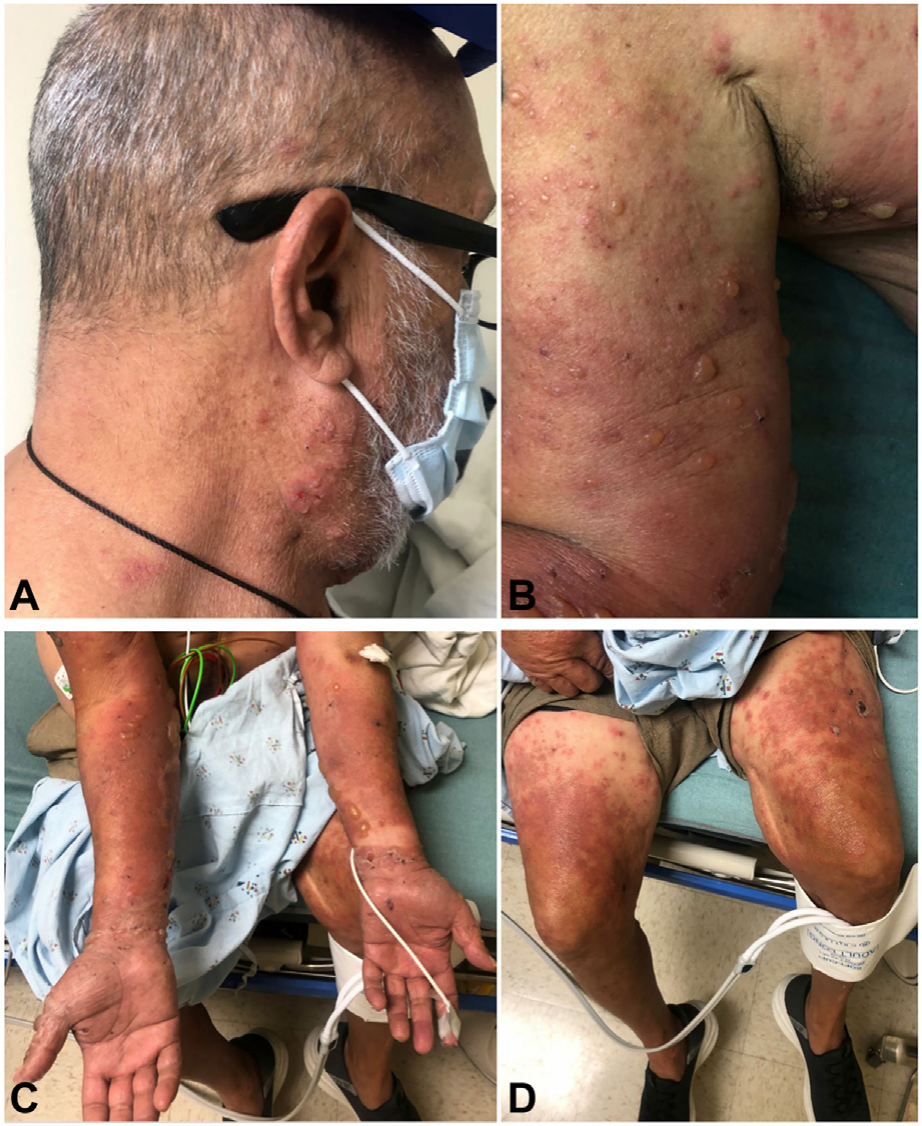
Exam findings with scattered tense vesicles and bullae on large erythematous plaques and few scattered weeping crusted erosions on the (**A**) scalp and neck, (**B**) *right arm*, (**C**) hands including palms, and (**D**) thighs.

A 64-year-old male with a history of vascular graft surgery, congestive heart failure, hyperlipidemia, and hypertension presented with a pruritic and tender blistering rash for 14 days. He had undergone vascular graft surgery 3 months prior and was started on doxycycline as prophylaxis for infection. He then returned to Mexico, where he lives and teaches surfing. Unaware of the risks associated with this medication, he did not use photoprotection despite long days in sun wearing only swim trunks, and developed a confluent red, pruritic rash in sun-exposed areas 1 month after starting doxycycline, thought to be phototoxicity. He was switched from doxycycline to amoxicillin-clavulanic acid and limited sun exposure. He subsequently developed diarrhea and discontinued amoxicillin-clavulanic acid after 3 weeks. Despite discontinuing antibiotics, the diffuse erythema persisted. Two weeks prior to hospitalization, the rash became painful and blistered. Dermatologic exam was notable for diffusely scattered tense vesicles and bullae on large erythematous plaques and few scattered weeping crusted erosions on the scalp, neck, upper torso, arms, hands including palms, and thighs to mid-calf (Fig 1, *A-D*). Mucosal involvement was not detected.

**Fig 2:**
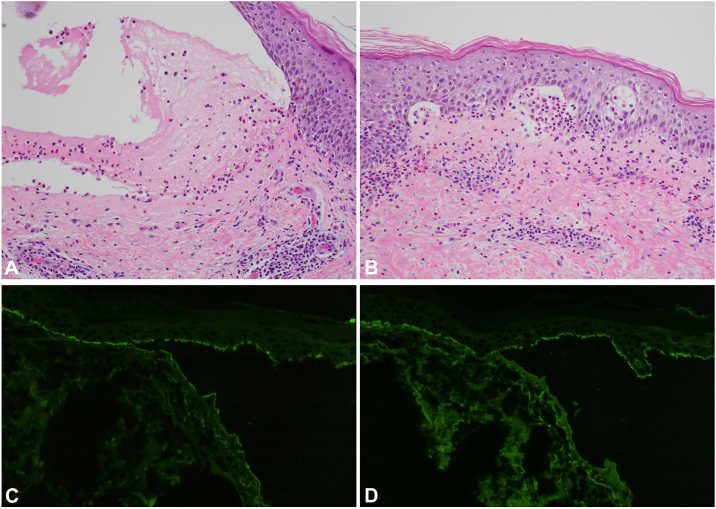
H&E and direct immunofluorescence on salt-split skin findings consistent with a diagnosis of bullous pemphigoid. **A,** H&E staining demonstrates a subepidermal split. **B,** mixed inflammatory cell infiltrate containing lymphocytes, histiocytes, and numerous eosinophils. **C,** linear deposition of complement component 3 along the roof and floor of the induced blister. **D,** linear deposition of immunoglobulin G along the roof of the induced blister. Salt-split was validated with collagen IV immunostain to confirm the tissue split correctly at the lamina densa. Original magnifications: **A,** 20×; **B,** 20×; **C,** 40×; **D,** 40×.

His other medications included metoprolol, aspirin, clopidogrel, and simvastatin, all of which had been stable for 2 years. Cilostazol was started one and a half months prior to rash onset. Biopsy of lesional skin for hematoxylin and eosin (H&E) and perilesional skin for direct immunofluorescence was performed on the upper portion of the right arm (Fig 1, *B*). H&E staining demonstrated a clean, subepidermal split (Fig 2, *A* and *B*) and a mixed inflammatory cell infiltrate containing lymphocytes, histiocytes, and eosinophils in the superficial dermis. Direct immunofluorescence demonstrated linear deposition of C3 and IgG along the basement membrane zone (BMZ), with a salt-split study showing linear deposition of IgG along the roof of the blister and linear deposition of C3 on the roof and floor of the induced blister (Fig 2, *C* and *D*). Indirect immunofluorescence was notable for positive IgG on monkey esophagus (1:640) and positive IgG on human split skin substrate (1:1280), while an enzyme-linked immunosorbent assay study demonstrated a positive IgG BP antigen 180 antibody level (106 U/mL; normal/negative: <9 U/mL) and negative IgG BP antigen 230 antibody level (6 U/mL), confirming a diagnosis of BP.

The patient was started on 1 mg/kg/day of by mouth prednisone and clobetasol ointment twice a day. After BP was confirmed, he was started on niacinamide 500 mg by mouth three times a day and IV immunoglobulin 2 g/kg over 4 days. Doxycycline was not initiated due to concern for this being an inciting factor. One day after starting IV immunoglobulin, he reported improvement of his symptoms, including itching and pain. The patient was continued on prednisone and niacinamide at the time of discharge and subsequently tapered off prednisone and started on 500 mg of mycophenolate mofetil twice a day. At 2-month follow-up he continued this regimen with no blister recurrence.

## Discussion

BP risk factors include age, specific systemic medications, UV radiation, and infections.^2^, ^3^, ^4^, ^5^ Medications associated with drug-induced BP include antibiotics, angiotensin converting enzyme inhibitors, dipeptidyl peptidase 4 inhibitors, non-steroidal anti-inflammatory drugs, salicylates, diuretics, immune checkpoint inhibitors such as programmed cell death protein 1 and programmed cell death-ligand 1 inhibitors, and others.^2^ Drug-induced cases of BP have been reported to occur both exclusively on sun exposed areas in which phototoxicity occurred,^4^ as well as on both sun exposed and sun protected areas.^5^ The majority of our patient’s lesions localized to sun-exposed areas, with minimal spread to partially sun-protected areas such as the scalp and palms. A proposed mechanism for the pathogenesis of drug-induced BP is the recognition of drugs as antigens that covalently interact with endogenous proteins to modify their antigenic properties resulting in the uncovering of hidden antigenic sites or the generation of new ones.^2^

Antibiotics were one of the first classes of medications associated with drug-induced BP, particularly in patients younger than those typically affected by BP.^2^ Among the antibiotics reported to have an association are penicillins, cephalosporins, quinolones, nitroimidazoles, and actinomycin. The mechanisms underlying antibiotic-induced BP are thought to relate to chemical structure. For example, thiol drugs may provoke direct damage to the DEJ via a nonimmunogenic mechanism, exposing new antigens to the immune system leading to synthesis and release of autoantibodies against BMZ proteins.^5^

The offending drug in this case was thought to be doxycycline, which is typically part of the multi-drug management of BP due to its anti-inflammatory action,^6^ yet known to induce phototoxicity.^7^ In turn, UV exposure has been reported to be an aggravator of existing BP, and cases of light-induced and phototoxicity-induced BP have been reported (Table I). The pathogenic mechanism of phototoxicity-induced BP may result from UV-mediated tissue disruption leading to exposure of antigens at the BMZ and development of autoantibodies.^2^ In the literature there are at least 14 cases of BP triggered by psoralen and ultraviolet A and ultraviolet B therapy for psoriasis and 4 cases for mycosis fungoides, but these were excluded from Table I given lack of clear documentation regarding phototoxicity. In the case of psoriasis, the exact triggering factor remains controversial and may include psoriasis-dependent BMZ changes and dysregulation of T-cell activity resulting in the induction of antibodies against BMZ antigens.^10^

**Table I:** Reported cases of phototoxicity-associated bullous pemphigoid

Reference	Inciting factor	Number of patients	Associated medication	Treatment
Pfau et al^3^	Sun exposure (UVA)∗	1	Unknown	Methylprednisolone
Takeichi et al^4^	Sun exposure (UVA)∗	1	Furosemide	Prednisolone, TCI
Rakvit et al^8^	Photodynamic therapy	1	N/A	TCS
Kluger et al^9^	Photodynamic therapy	1	N/A	Prednisolone, TCS

*TCI*, Topical calcineurin inhibitor; *TCS*, topical corticosteroids; *UVA*, ultraviolet A.

∗ As determined by subsequent provocation testing with ultraviolet A.

Based on the timing and sequence of events and the photo-distributed nature of his lesions, we believe that doxycycline-induced phototoxicity played an integral role in the development of BP in this patient. We hope this case promotes awareness of the possible association of doxycycline, a known BP treatment, and the onset of BP via its potential to cause phototoxicity.

## Conflicts of interest

None disclosed.
